# Dynamic livelihoods, gender and poverty in marine protected areas: Case study from Zanzibar, Tanzania

**DOI:** 10.1007/s13280-024-02010-x

**Published:** 2024-04-22

**Authors:** Felicity Pike, Lars Lindström, Josefin Ekstedt, Narriman S. Jiddawi, Maricela de la Torre-Castro

**Affiliations:** 1 Department of Physical Geography, Stockholm University, Stockholm, Sweden; 2 Department of Political Science, Stockholm University, Stockholm, Sweden; 3 Centre for Blue Governance, Department of Sustainability and Planning, Aalborg University, Aalborg, Denmark; 4 Zanzi Marine and Coastal Solutions, P.O. Box 4108, Chukwani, Zanzibar Tanzania

**Keywords:** Rural development, Seaweed farming, Small-scale fisheries, Tourism, Livelihood transitions, East Africa

## Abstract

Livelihood initiatives are common within marine protected areas (MPAs) aiming for poverty alleviation or higher income opportunities. However, results can be mixed in reality, as well as change over time. Furthermore, *who* benefits is a key consideration, as results can vary based on inequalities, including gender. Here, the monetary outcomes of different livelihood strategies were investigated across three MPA regions in Zanzibar, Tanzania. Using a quantitative approach, the results show that livelihoods have shifted in a six-year period, with livelihood strategies differing in poverty incidence and income. Livelihood initiatives, namely seaweed farming and tourism, did not provide significantly higher monetary returns compared to long-standing livelihoods, such as fisheries. Seaweed farming showed income stability but a high poverty incidence predominantly within women-headed households. During the study period, men primarily remained in fisheries, whilst women shifted to small-scale businesses and fisheries, largely exiting seaweed farming. This underscores a need for adaptive, gender sensitive management within fast changing coastal contexts.

**Supplementary Information:**

The online version contains supplementary material available at 10.1007/s13280-024-02010-x.

## Introduction

Within coastal zones, livelihood interventions represent key actions taken to reduce the decline of natural resource bases and enable positive outcomes, such as higher income and poverty alleviation (Allison and Ellis 2001). At their core, livelihoods can be understood as the means by which people make a living, including activities and assets (Scoones 2009). Importantly, livelihoods often involve a diversity of activities, meaning that understanding how the combination of activities (i.e. livelihood strategies) produces outcomes at various scales can be highly informative, as opposed to focusing on single activities (Scoones 2009). Furthermore, within coastal zones, understanding how present livelihood situations have emerged or shifted over time is important due to the changeable nature of these environments (Steenbergen et al. 2017; Trung Thanh et al. 2021). Coastal livelihood interventions target various aspects, including mechanisms that alleviate persistent poverty (e.g. Brugère et al. 2008; Roscher et al. 2022b). Poverty can be viewed in absolute terms (situated below an income or expenditure threshold) and multidimensional terms which encompasses income, alongside deprivation across other domains, such as education and infrastructure (World Bank 2018). More intangible aspects such as power or psychological domains may also be included as multidimensional terms (Kakwani and Silber 2013; Vijaya et al. 2014).

Poverty alleviation is increasingly visible within natural resource management and conservation contexts, such as marine protected areas (MPAs) (Nowakowski et al. 2023). These holistic approaches follow a growing emphasis for protected areas to meet the needs of communities to enable effective conservation (Maxwell et al. 2020). Nonetheless, positive social outcomes from MPAs, including poverty alleviation and successful livelihood interventions, can be elusive and mixed in the extent to which they benefit people (Westlund et al. 2017; Ban et al. 2019). Reasons for successes or failures are not always clear. Thus, furthering understanding of whether social benefits are or are not occurring for different social groupings (as opposed to the community in general), alongside reasons for why this might be, is needed across different contexts to pave a more effective path forward.

Examples of livelihood interventions that are commonly seen within MPAs include diversification and generation of novel livelihood opportunities, with employment in tourism being a typical example (e.g. Pham 2020; Tranter et al. 2022). Livelihood diversification can be a relatively ambiguous term that is often associated with adding supplementary activities to existing individual or household livelihood portfolios, as well as diversifying strategies and assets used within livelihoods, or introducing new activities that serve as alternative livelihoods (Roscher et al. 2022b). These initiatives may provide win–wins through reducing pressures on natural resources and enable flexibility towards stressors thus helping to reduce vulnerabilities and *potentially* break poverty traps (Haider et al. 2018). They may also be a means of compensating lost income from fishery regulations imposed by MPAs (Katikiro 2016; Pham 2020). Furthermore, interventions may generate community support for MPAs through buy-in mechanisms which can help build trust and compliance (Tobey and Torell 2006). The materialisation of positive outcomes from these livelihood interventions, which can be exogenous (externally induced) or endogenous (self-induced), is often assumed, yet when empirically tested, outcomes can be highly mixed (Roscher et al. 2022a). Importantly, introducing diversified and alternative sources of income does not necessarily translate to exits from resource extractive activities, such as fishing (de la Torre-Castro and Lindström 2010; Cinner 2014; Katikiro 2016).

Gender divisions in livelihood activities are well documented in coastal and marine spaces (Koralagama et al. 2017; de la Torre-Castro et al. 2017). Nonetheless, consideration of gender differences within MPA and livelihood initiatives often receives relatively little attention, despite potentially significant roles in achieving positive and equitable outcomes (Barreto et al. 2020). Furthermore, studies on gender and poverty in general within coastal livelihood contexts are limited compared to terrestrial counterparts. Specifically, there is a need to further our understanding of how gender differences affect the experience and alleviation of coastal poverty (Lawson et al. 2012; Stacey et al. 2019). From a livelihoods perspective, gender norms, roles and expectations can be central to the differences by which people can access, engage with and have control or choice over livelihoods, including new opportunities built from initiatives (de la Torre-Castro et al. 2017; Lawless et al. 2019; Barsoum 2021). Gender differences in livelihoods, as well as the social constructs that underlie them, are dynamic and can vary greatly across space and time (Barsoum 2021).

Here, a case study in Zanzibar, Tanzania, was conducted with three main objectives. Firstly, the study addresses what changes in livelihoods can be observed for men and women across MPA regions in a 6-year period? Secondly, regarding overall household livelihood strategies—how do monetary outcomes and poverty incidence differ across strategies? Thirdly, what factors are linked with pursuit of these strategies? In particular, does the gender of the household heads or MPA region in place have significance? Livelihoods that relate to forms of intervention and sustainable development initiatives were of special interest. Specifically, whether such livelihoods offered higher monetary returns compared to more longstanding livelihoods such as fishing. The case study area, Zanzibar, constitutes a tropical archipelago within a lower-middle income region. Reliance on marine and coastal livelihoods is high, and coastal poverty is a persistent problem (World Bank 2022). Zanzibar is a representative context from which parallels can be drawn from, particularly across tropical island contexts.

Within the study, six livelihood sectors were focused upon including two which can be described as arisen from sustainable livelihoods initiatives and interventions. These were aquaculture, which in Zanzibar constitutes seaweed farming as the only sector at commercial scale (Charisiadou et al. 2022) and tourism. Tourism is encouraged by MPAs as well as being a priority sector within emerging Blue Economy policies and initiatives that aim to expand the economic potential from marine and coastal environments within sustainable limits (Zanzibar Planning Commission 2020). Both aquaculture and tourism are focal points for livelihood interventions within Zanzibar MPAs, with both being possible means of livelihood diversification (Gustavsson et al. 2014; Torell et al. 2017). In the case of aquaculture, seaweed farming constitutes a priority for local politicians, and external donors to the MPA system, as a means of generating income and economic empowerment for coastal women specifically. The activity has also been a priority point in the Zanzibar Strategy for Growth and Reduction of Poverty (MKUZA) as a means of livelihood diversification (McLean et al. 2012). Nonetheless, differing opinions exist on the effectiveness of seaweed farming in these regards (e.g. Msuya 2011; Fröcklin et al. 2012; Torell et al. 2017). Accordingly, comparison of aquaculture and tourism livelihoods were of particular interest in this study in terms of their monetary returns and poverty implications in comparison with traditional fishery and land resource-based livelihoods. Importantly, the study focuses primarily on the monetary outcomes of livelihoods, with income being the primary point of analysis. The reasoning for focusing primarily on income is that income forms a direct and tangible outcome from activities, with a clear role in poverty incidence and why people may benefit from or pursue these activities. In addition, income has a role in building economic power for a higher quality of life, through increased purchasing power and greater agency for decision-making—this, overall, can promote freedom and emancipation which may in turn lower more relative domains of poverty related to these domains (Kabeer 1999).

## Materials and methods

### Study site and MPA overview

Zanzibar comprises an archipelago region located 40 km off mainland Tanzania within the Western Indian Ocean. The archipelago constitutes 50 islets and two larger islands, the largest of which is called Unguja. Unguja is the location of the study and will be referred to as Zanzibar hereafter. Zanzibar is semi-autonomous, meaning that the region has autonomy and self-governance over numerous matters, whilst being within the overall Republic of Tanzania. The marine and coastal environment of Zanzibar is diverse, with a seascape comprising of productive habitats including coral reefs, seagrass meadows, mangroves and macroalgae (Yahya 2021). With a tropical climate, Zanzibar undergoes two annual monsoon seasons between April–September, the southeast monsoon, and November–February, the northeast monsoon (Yahya 2021).

**Fig. 1:**
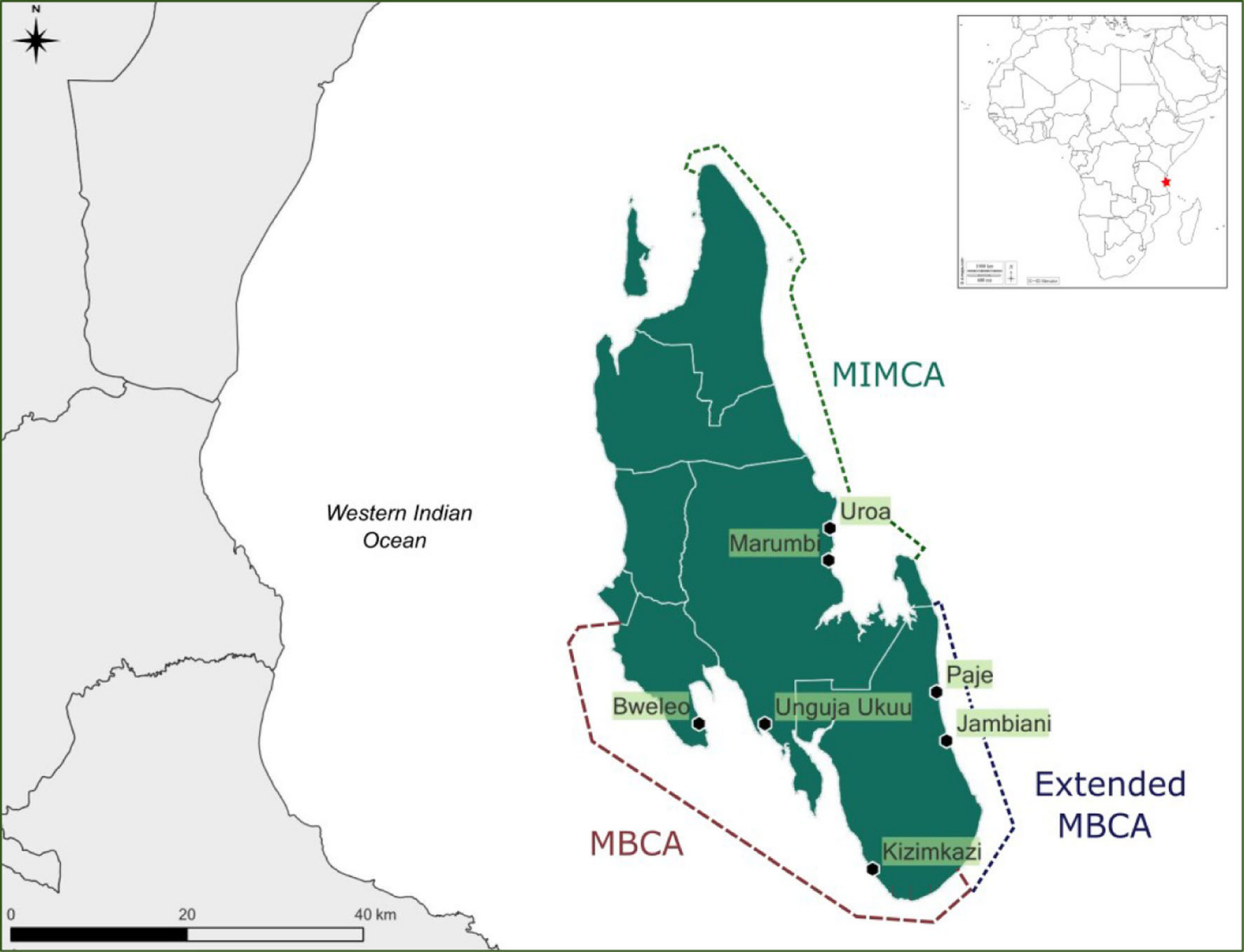
Location of study sites and boundaries for the three marine protected area regions—the Menai Bay Conservation Area (MBCA), the extended boundary for the MBCA and the Mnemba Island—Chwaka Bay Conservation Area (MIMCA). The MBCA currently covers 717.5 km^2^, and MIMCA 337.3 km^2^. (Map sources: Zanzibar – qGIS with district shapefiles from Tanzania National Bureau of Statistics. Overlay - https://d-maps.com/carte.php?num_car=737&lang=en)

Regarding MPAs, implementation has been extensive across most of the coastline. Within this study, two MPAs, as well as an extended boundary of one of these MPAs were investigated (three MPA regions in total). These were the Mnemba Island—Chwaka Bay Marine Conservation Area (MIMCA), the Menai Bay Conservation Area (MBCA) and the extended boundary of the MBCA which occurred in 2014 (Fig. 1). The MBCA is the oldest (implemented in 1997) and largest MPA in Zanzibar, constituting a general-use zone with more enforcement of fishing regulations than other MPAs—although enforcement is generally still low (Colbert-Sangree and Suter 2015). Tourism in the MBCA is frequently given as a community benefit to those residing within the MPA, with ecotourism sectors such as dolphin tours which MBCA has the authority to impose regulations upon (McLean et al. 2012). Tourism benefits include both revenues going into communities, as well as livelihood opportunities (Lange and Jiddawi 2009). MIMCA (implemented in 2002) is also mostly a general-use zone with regulations on fishing activities, but enforcement has been described as very low (de la Torre-Castro and Lindström 2010; Gustavsson et al. 2014). A small no-take zone (< 1 km) does exist around the Mnemba Island atoll which is enforced and coral recovery has been reported (McLean et al. 2012). Tourism opportunities exist within beach resorts throughout the MPA, as well as mangrove ecotourism within Chwaka Bay (managed by the separate Jozani Chwaka Bay National Park). As Zanzibar’s MPAs largely focus on sustainable use of natural resources, there are numerous forms of direct engagement in livelihoods that may impact residing communities, e.g.

1. Establishing stakeholder engagement mechanisms, such as seaweed farming committees (McLean et al. 2012).
2. Enabling opportunities for tourism development, e.g. through dolphin conservation in the MBCA or the no-take zone of the Mnemba atoll in MIMCA which is a diving hotspot (Pike et al. 2022).
3. Supporting alternative livelihood initiatives, such as aquaculture and land-based activities (Tobey and Torell 2006).
4. Supporting development of fishery infrastructure, including providing cooling facilities in landing sites, gear exchange schemes and fibre boats in some cases (Tobey and Torell 2006).

### Livelihood overview

Both historically and presently, a large proportion of the population engages in marine and coastal related livelihoods, particularly within small-scale fisheries (Jiddawi and Öhman 2002). Small-scale fisheries largely use traditional methods, although with modernisation of gears and vessels in some cases, e.g. motorised boats. Men and women usually use different fishing techniques, with men tending to engage in fin-fisheries or higher value invertebrate fisheries—often in deeper water, with diverse gears (de la Torre-Castro et al. 2017). In parallel, women tend to pursue gleaning—which involves collecting invertebrates at low tide with little-to no gear (Nordlund et al. 2010). Women also form the majority of the seaweed farming demographic (Charisiadou et al. 2022). The activity quickly became widespread during the 1990s, particularly amongst women (Msuya 2011). Seaweed farming constitutes off-bottom cultivation of the non-native species’ *Kappaphycus alvarezii* and *Eucheuma denticulatum* within the intertidal zone. Commercial seaweed farming was introduced in 1989, with funding from buying companies, as well as support from development organisations, such as USAID (Pettersson-Löfquist 1995). The idea originated from researchers following visits to established farms in the Philippines, with particular interest in its low environmental impact, as well as positive possibilities for income generation (Bryceson 2002). Over time, seaweed farming has raised concerns in terms of poor working conditions and low productivity (Fröcklin et al. 2012; Makame 2022; de la Torre-Castro et al. 2022), vulnerability to climate change (Hassan and Othman 2019; Matoju et al. 2022) and negative impacts on macrobenthic habitats (Eklöf et al. 2005; Moreira-Saporiti et al. 2021).

Tourism is now Zanzibar’s leading economic sector following rapid sectoral growth since the 1990’s (Khamis et al. 2017). Coastal and marine-based tourism entails significant macroeconomic contributions to the region and includes a diversity of livelihoods both within formal employment (e.g. diving operators, blue safaris and hotels) and the informal sector (e.g. small-scale businesses targeting the tourism market). The strains of expanding tourism infrastructure on coastal ecosystems has been problematic in cases, as well as uneven distribution in benefits and costs—e.g. livelihood displacement from restricted coastal access and little revenue reaching communities (Lange 2015). Agricultural livelihoods include animal husbandry, forestry and cultivation (often smallholdings) with important cash crops including cloves, coconut, spices, rice and various fruits (Khamis et al. 2017). An example of a common forestry livelihood is firewood collection within coastal forests and mangroves, with this typically being participated in by women (de la Torre-Castro et al. 2017). Further livelihood opportunities include various roles within the informal sector through small-scale businesses (e.g. textiles, vending, etc.), skilled trades (e.g. welding, carpentry, etc.) and casual employment (e.g. within construction, transport, shops, etc.).

### Household surveys

All data were collected from household surveys conducted within the three MPA regions during two time periods: 2013/2014 (*n* = 217) and 2019/2020 (*n* = 165) (Table 1). During the 6-year interval between the two survey periods, the 2014 extension of the MBCA occurred—therefore two sites, Paje and Jambiani, were not within MPA boundaries during the 2013/2014 surveys, but then had been incorporated into the MBCA by the time of the 2019/2020 surveys. By the start of the 2019/2020 period, Paje and Jambiani had been within the MBCA for over 5 years. The surveys were conducted by random sampling households within the different villages. The surveys were conducted in Swahili with assistance from translators whom had received training in survey techniques (with a researcher always present). A minimum of 40 surveys were conducted in each MPA region. No specific exclusion criteria applied. The survey consisted of structured questions relating to which livelihood activities were being pursued by different household members. Information on household characteristics was also gathered (see Table S4). Heads of households were the respondent when possible, if not another household member (> 18 years) was asked with this usually being a spouse. Activities could include those pursued anywhere, including outside MPA boundaries. Pilot surveys were conducted in both time periods prior to formal data collection. Informed consent was obtained through presenting the study’s purpose and how the data were to be used beforehand. Respondents were able to end the survey and have their responses deleted at any time.

**Table 1:** Numbers of surveys conducted in each study area during each time period (2013/2014 and 2019/2020)

MPA region	Sites included	Number of surveys conducted in 2013/2014	Number of surveys conducted in 2019/20
Mnemba Island—Chwaka Bay marine conservation area (MIMCA)	Marumbi, Uroa	71	60
Menai Bay conservation area (MBCA)	Unguja Ukuu, Bweleo, Kizimkazi	78	58
The MBCA extension	Paje, Jambiani	68	47
		*N* = 217	*N* = 165

This includes two MPAs (MIMCA and MBCA), as well as an extended area of the MBCA which occurred in 2014. Interviews in the MBCA extension were done before and after the extension formally took place

The surveys obtained information on livelihoods at both the individual and household scale, described as follows:

1. Individuals livelihood changes.
  Individual livelihoods were observed as the main livelihood activity being pursued by the heads of the households and second main income earner, usually a spouse, when applicable. This information was obtained in both survey periods (i.e. 2013/2014 and 2019/2020) and used as the basis for the time comparison. By focusing on the two main income earners, this allowed for a wide-scale comparison, as it encompasses how most households are structured. The aim here was to understand how individuals’ livelihoods have changed in the 6-year interval across MPAs, and between men and women.
2. Household livelihood strategies.
  In the 2019/2020 surveys, all livelihood activities pursued by all adults in the household were recorded, along with the income associated with these activities. This formed the basis of the cluster analysis (described in the next section) and identification of household strategies. These strategies differ from individuals’ livelihoods in that they encompass the entirety of activities pursued in the household to generate income. Surveys from 2013/2014 were only used to assess how livelihoods have changed over time, and not used within this analysis. This is due to information on income derived by different activities in the households not being available from these surveys; therefore, cluster analysis or strategy identification was not done with these surveys.

### Cluster analysis: Defining livelihood strategies

Clustering households into groups containing similar income generating characteristics is a longstanding approach within livelihood studies (e.g. Jansen et al. 2006; Soltani et al. 2012). The eventual clusters from this type of analysis can be considered the livelihood strategies common to the surveyed households. To categorise livelihood groupings from the 2019/2020 in survey responses for further analysis, thirty sub-categories (Table S1) were defined and further categorised into six sectors: (i) fisheries, (ii) aquaculture, (iii) agriculture, forestry and livestock, (iv) tourism, (v) salaried public sector, (vi) informal sector and (vii) other. Livelihoods were categorised into these final sectors through characteristics described in Table S2. In particular, tourism livelihoods were categorised based on employment within the sector. Small-scale businesses which may be aiming at the tourist trade were categorised under the informal sector.

The study used principle component analysis (PCA), and then hierarchal cluster analysis (HCA) to define distinct livelihood clusters (or strategies) based on similarity in percentage income generated from the six described livelihood clusters. PCA analysis was done on the percentage contributions from each livelihood group to the total income of 165 households. Factor loadings from the principle components with eigenvalues greater than 1 were used as input data for HCA following the Ward method. The HCA was followed by k-means clustering to further distinction of the eventual clusters and lower risks of misclassifications. The output entailed categorisation of the households based on similarities in the sources of income based on the livelihood groups. Significant differences in income share amongst the clusters were tested with one-way analysis of variance (ANOVA) and post hoc Tukey’s test.

### Poverty and monetary return analysis

To assess difference in monetary returns across the eventual livelihood strategies, per capita income (i.e. total household income divided by the number of household members) was used as a proxy. Significant differences in per capita income across the livelihood clusters were tested with a Kruskal–Wallis and post hoc Wilcoxon’s test due to non-normal distribution. To assess poverty incidence, three approaches were taken: first-order stochastic dominance, a multidimensional poverty measure and a headcount ratio. First-order stochastic dominance amongst the livelihood clusters was evaluated by plotting the cumulative distribution function of per capita income across the households categorised within each strategy. Stochastic dominance is a method of ranking variables based on cumulative distributions, which can be useful within poverty assessments, e.g. through identifying groups, livelihoods or strategies as higher returning in relation to others (Paudel Khatiwada et al. 2017; García-Gómez et al. 2019). A livelihood cluster can be classified as stochastically dominant if it consistently ‘outperforms’ another based on the distribution of households earning within the range of income levels across the dataset.

Alongside stochastic dominance, poverty incidence in households amongst strategies was assessed through two measures: the World Bank Multidimensional Poverty Measure (MPM) (World Bank 2018) and a headcount ratio (i.e. percentage of households earning below the international poverty line within each strategy). The MPM constitutes a weighted index that allows for inclusion of relative domains of poverty beyond monetary deprivation, including education and access to basic infrastructure. These domains are assessed through weighted binary indicators (Yes/No) to produce a 0–1 scalar index (see Table S3). An MPM index of 1 would indicate high multidimensional poverty on the basis of deprivation across all domains. Alternatively, the headcount ratio provides an indication of how many households pursuing different strategies are in absolute poverty circumstances based on earning below the international poverty line (1.9 USD during data collection in 2019/2020).

### Household characteristics linked to livelihood strategies

To assess whether the likelihood of different livelihood strategies being pursued changed across MPAs and certain household characteristics, multinomial logistic regression was used. These household characteristics used as explanatory variables were the gender, age and education level of the head of household, as well as the MPA situation in place. For specifics on indicators used for each characteristic, see Table S4. Regarding gender of the household heads, in total there were 134 men-headed households compared to 31 women-headed households within the survey sample. Livelihood strategies, i.e. the clusters, were used as the response variable. Multicollinearity was checked through inspection of variance inflation factors (VIFs), and independence of irrelevant alternatives was checked through the Hausman–McFadden test. All analyses were performed in R version 4.1.0 (R Core Team 2021) with packages.

## Results

### Livelihood changes

**Fig. 2:**
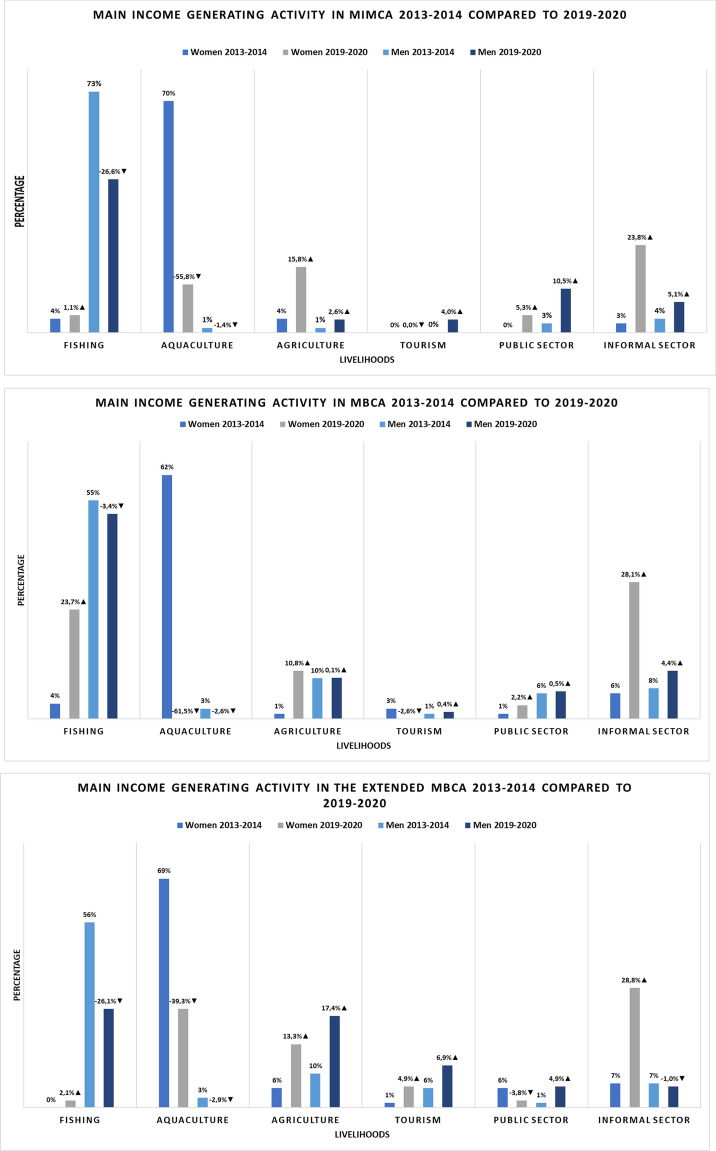
Main livelihood changes for men and women between the 6-year period 2013/2014 and 2019/2020 across three MPA regions. These are the Menai Bay Conservation Area (MBCA), Mnemba Island Chwaka Bay Conservation Area (MIMCA) and the extended MBCA. The extended MBCA constitutes two sites (Paje and Jambiani) which were outside of MPA boundaries in 2013/2014 and then part of the MBCA as part of the boundary extension by the time of 2019/2020. Main livelihoods included income-generating activities only, and not, for example, subsidence fishing or farming

Changes to men and women’s livelihoods were observed between the time period 2013/2014 and 2019/2020 across the three MPA regions (Fig. 2). This showed several apparent changes. Overall, there was a clear trend of exits from women in aquaculture livelihoods, which largely constitutes seaweed farming. During 2013/2014, aquaculture appeared to be the dominating main livelihood for women across all three MPA regions. This had then shifted to the informal sector being the main livelihood in 2019/2020. Women appeared to also shift to agricultural livelihoods across the three MPAs, and then, within the MBCA, there was also a large increase in women engaging in fishery livelihoods. Regarding men, fishery livelihoods remained the dominant livelihood in both time periods, although fishery exits were apparent, particularly within MIMCA and then minimally within the MBCA. Across the MPAs, tourism as a main livelihood remains to be for a low proportion (< 10%) of respondents. Tourism was more commonly a main livelihood for men (most so in MIMCA where no women had tourism as a main livelihood). Tourism livelihoods have been increasing in prevalence slightly across the MPAs—most so in the extended MBCA region.

### Identifying common household livelihood strategies

Four distinct clusters were identified from the HCA—and named based on the livelihood sectors which had dominating income shares. This was defined by a sector contributing over 20% to the total household income. These clusters were (1) fisheries; (2) tourism; (3) Aquaculture/other—i.e. remittances and pensions and (4) mixed land-based strategies which included income from the informal sector as well agriculture, livestock and forestry. Tourism livelihoods were defined as employment, either formal or casual, within tourism work, thus not including small-scale businesses that may be targeting the tourist trade (either directly or opportunistically. These activities were assigned to the informal sector. These clusters can then be described as household livelihood strategies. Numbers of households pursuing each strategy differed, with fisheries being the most common (*n* = 86) and aquaculture/other (*n* = 10) being least common. Household per capita income across strategies also significantly differed according to a Kruskal–Wallis test (*p* = 3 0.27e−07) (Table 2). A post hoc Wilcoxon test (Table S6) was used to derive pairwise comparisons, with significant differences found between each strategy pair except fisheries and tourism and tourism and mixed land-based.

**Table 2:** (A) Final clusters from the hierarchal clustering analysis (HCA) on sectoral income share. Loadings from the factor analysis (Table S5) were used as input to the HCA

		**A**							**B**	
		Mean percentage share of total sectoral income (% (± SD))							Average daily per capita income (USD)	
Cluster	Orientation	Fisheries	Aquaculture	Agriculture, Forestry and Livestock	Tourism	Informal Sector	Public Sector	Other	Mean	± SD
1 (*n* = 86)	Fisheries	**81.71 (17.62)**	1.35 (4.83)	2.92 (6.73)	1.29 (5.88)	8.92 (13.67)	3.04 (9.32)	0	3.19	3.08
2 (*n* = 14)	Tourism	9.91 (17.07)	2.8 (4.34)	6.94 (13.48)	**73.49 (13.98)**	6.85 (13.98)	0	0	3.32	3.68
3 (*n* = 10)	Aquaculture/other	0	**21.78 (35.29)**	3.1 (9.81)	0	3.33 (10.54)	0	**71.79 (39.92)**	0.15	0.08
4 (*n* = 55)	Mixed land-based	6.01 (11.92)	1.49 (5.17)	**24.63 (33.52)**	0.32 (2.12)	**48.75 (38.67)**	17.64 (31.81)	0.2 (0.89)	2.37	2.98

Mean percentages of each livelihood sector to the total household income are shown, with standard deviation (SD) shown in brackets. Cluster orientations were assigned based on which sectors, highlighted in bold, had high percentage shares (> 20%) to the total household incomes. (B) Average daily per capita income (USD) across the livelihood clusters. Similarly, SD is shown in brackets. Differences in per capita income between clusters were significant (Kruskal–Wallis: *p* = 3.27e−07)

### Poverty and monetary returns across livelihood clusters

**Fig. 3:**
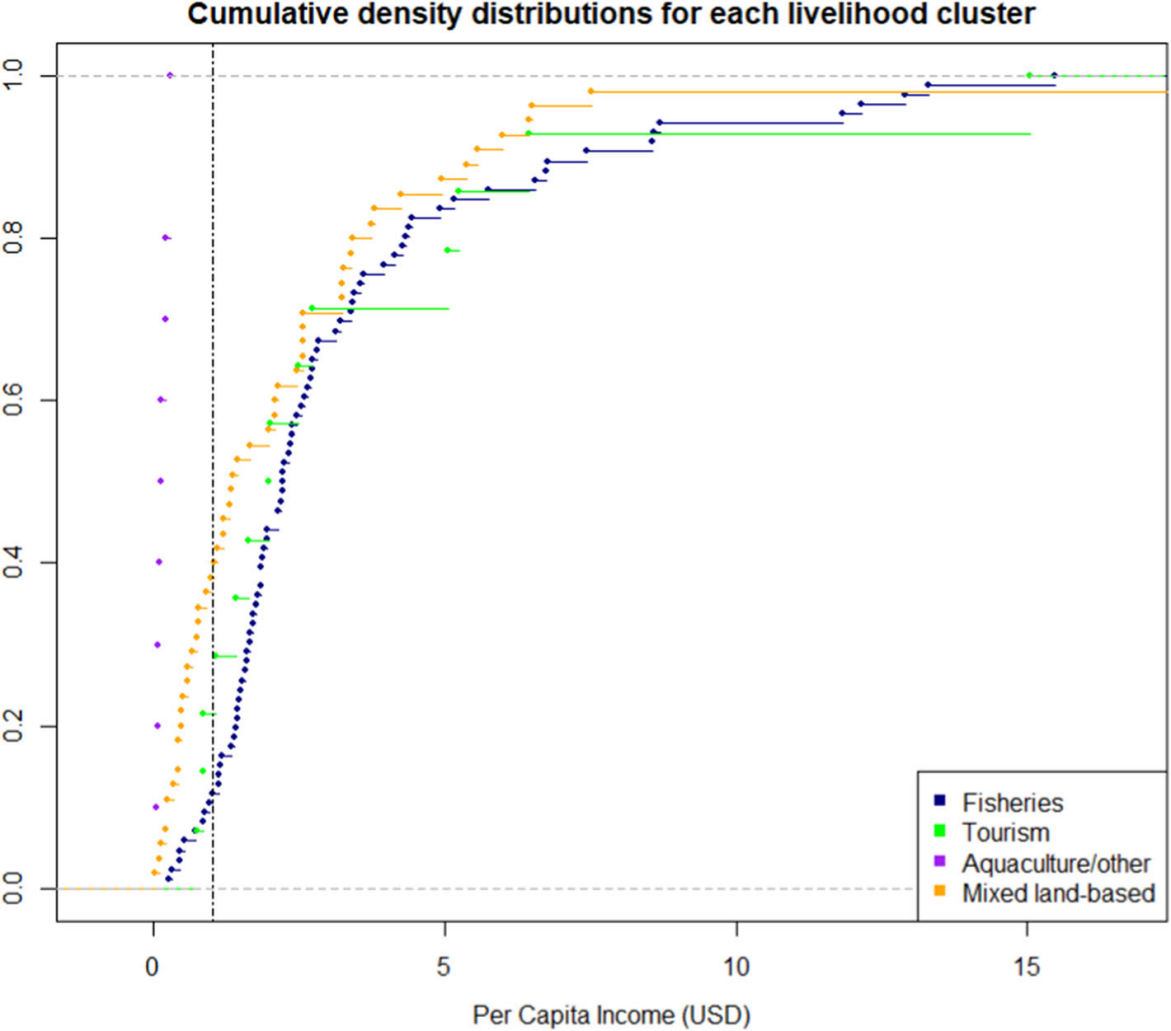
Cumulative density distributions for per capita income within each livelihood cluster/strategy. The intersect line represents the international poverty line (1.9 USD)

The four livelihood strategies demonstrated levels of difference in monetary returns, and proportions of households earning under the World Bank international poverty line ($1.9 per day) (Table 3). Additionally, difference in the multidimensional poverty measure was significant across strategies (Kruskal–Wallis: *p* = 0.0001) (Table 3). Significant pairwise differences were not found between the fisheries, tourism and mixed land-based strategies. Instead, the difference was accounted for, pairwise, by the aquaculture/other strategy and each of these other strategies. Households doing this strategy were also universally earning under the poverty line and were stochastically dominated by all other strategies (Fig. 3). This would indicate higher monetary and non-monetary deprivation compared to households pursuing other strategies. However, mean income within the strategy had a comparatively low standard deviation (*µ* = 0.15, SD ± 0.08). Overall then, monetary returns from the strategy could be described as very low, but relatively consistent.

**Table 3:** Poverty measures across the livelihood clusters

Cluster	Multidimensional poverty measure (Range 0–1)		Headcount ratio (households earning below 1.9 USD per capita)	
	Mean (± SD)†	Mode	*n* below 1.9 USD	Percentage of total (%)
1 (*n* = 86)	0.25 (0.18)	0.22	36	41.86
2 (*n* = 14)	0.24 (0.18)	0.44	6	42.86
3 (*n* = 10)	0.51 (0.11)	0.55	10	100
4 (*n* = 55)	0.28 (0.19)	0.44	30	54.55

The multidimensional poverty measure refers to the World Bank measure entailing three weighted domains: monetary, education and access to basic infrastructure. The measure ranges between 0 and 1, with a value of 1 indicating highest multidimensional poverty based on deprivation across all domains. Households earning below 1.9 USD per capita provides the percentage of households within clusters earning under the World Bank international poverty line, thus providing an indication of absolute poverty occurrence across the clusters

^†^Kruskal–Wallis (one-way analysis of variance): *p* = 0.0001

The tourism strategy had the highest per capita income (*µ* = 3.32, SD ± 3.68), although this was not significant except for when compared to aquaculture/other. The strategy also did not stochastically dominate either fisheries or mixed land-based strategies. The tourism mean per capita income was lower than these other two strategies, although not significantly according to the pairwise comparisons. The income distribution fluctuated and standard deviation was high (± 3.68), indicating comparatively high and low earners within the group, and a potentially less income-stable strategy. Multidimensional poverty was not significantly different to other strategies, except aquaculture/other (Table S7).

The fisheries strategy, which was the strategy most commonly pursued by households, stochastically dominated the aquaculture/other and mixed land-based strategies, but not tourism. The headcount ratio was lowest in this strategy although multidimensional poverty was not significantly lower except for in comparison with the aquaculture/other strategy (Table S7). Average per capita was highest in this strategy, although again standard deviation (*µ* = 3.19, SD ± 3.08) was high indicating that income was not consistently high and may not be very stable.

Overall, the fishery strategy could be reasonably described as a higher returning strategy, with lower absolute poverty incidence but not necessarily in respect to multidimensional poverty. Incomes were not consistently high in the strategy, and households are not monetarily better off than those pursuing tourism strategies according to the income distributions.

Finally, the second most pursued strategy was the mixed land-based strategy which appeared to have the most sectoral diversity through high income shares from the informal sector, public sector and livelihoods in agriculture, livestock and forestry. This strategy was stochastically dominated by fisheries, but not tourism. As with other strategies, it did stochastically dominate the aquaculture/other strategy. Average per capita income was the third ranking, again with a high standard deviation (*µ* = 2.37, SD ± 2.98). Incidence of multidimensional poverty was similar to the fisheries and tourism strategies (0.28); however, a greater proportion of households were earning below 1.9 USD compared to these two strategies.

### Household characteristics linked to livelihood strategies

Multinomial logistic regression was done with livelihood strategies as the response variable and different household characteristics as explanatory variables (Table 4). The fisheries strategy was used as the reference category, being the strategy mostly commonly pursued. Accordingly, in comparison with fisheries, the likelihood of households pursuing the aquaculture/other strategy increased with age of the household head (*β* = 0.09, *p* = 0.002), as well as when household heads were women (*β* = 3.7, *p* = 0.004). In regard to MPAs, compared with the original boundaries of the MBCA, household within the extended boundary was more likely to pursue tourism strategies (*β* = 1.97, *p* = 0.0). No other significant links were found with the MPAs. None of the household characteristics were also linked to likely pursuit of the mixed land-based strategy (compared to fisheries).

**Table 4:** Probabilities of household characteristics in predicting livelihood strategies in a multinomial logistic regression model

	Livelihood strategies					
	Tourism		Aquaculture/other		Mixed land-based	
Household characteristics	Coef.	SE	Coef.	SE	Coef.	SE
Intercept	− 0.42	1.51	− 7.95**	3.19	− 1.68*	0.93
MPA situation: MIMCA	− 0.43	0.95	− 0.40	1.53	0.11	0.42
MPA situation: Extended MBCA	1.97**	0.76	0.84	1.66	0.60	0.47
Household Size	− 0.13	0.20	− 0.16	0.32	0.12	0.11
Gender	0.56	0.85	3.70**	1.29	0.61	0.54
Age	− 0.04	0.03	0.09**	0.04	0.00	0.02

The fisheries strategy was used as the reference strategy. Coefficients (Coef.) and standard error (SE) are presented along with indication when a household characteristic was significant in predicting the strategy. Household head education was removed due to multicollinearity

*Significant at 10%; **Significant at 5%

Pseudo-R-squared: McFadden = 0.2; Cox and Snell = 0.33; and Nagelkerke = 0.37

Log likelihood: **− **141.37

Goodness of fit: Pearson chi-squared = 71.81 (*p* = 0.00)

## Discussion

This study involved an assessment of livelihoods at individual and household scales, focusing on monetary outcomes and poverty incidence as tangible benefits from people pursuing various activities or strategies. In order to gain understanding of how these livelihoods may be changing, the study also observed how gender-disaggregated livelihoods have changed across a 6-year period in Zanzibar’s MPAs. Changes may reflect evolving gender norms as well as systemic changes in the environment, economy and political backdrop. During the 6 years, women’s main livelihoods had shifted largely away from aquaculture (which mostly involves seaweed farming of red *Eucheuma* spp.) across the MPAs, but especially within the MBCA. Common destinations from these seaweed farming exits were fishing, the informal sector (e.g. small-scale business) and agriculture, forestry and livestock. The overall large exits from individual’s seaweed farming seen in this study would suggest the increasing unviability of seaweed farming as a livelihood, e.g. due to climate change as previously documented (Hassan and Othman 2019; de la Torre-Castro et al. 2022). Alternatively, other livelihoods which better serve needs are within reach to women—which would indeed be positive. For instance, shifting from climate vulnerable livelihoods, such as seaweed farming, into small-scale business has been described very positively (Makame et al. 2023). If these small-scale businesses are effectively utilising the tourism trade, this can also serve as a way for women to capture some of the benefits associated with the sector, even if employment opportunities remain scarce. However, doing this shift requires capital, particularly in the form of social and financial assets—which creates entry barriers (Makame et al. 2023). Furthermore, possible issues can arise from high supply over demand when too many are in the same market. Entrepreneurial solutions, such as small-scale businesses, can be effective poverty alleviation measures, particularly when combined with robust microfinance mechanisms, such as short-term credit (Bruton et al. 2013; Banerjee et al. 2019). This could be a worth avenue to explore as a form of institutional support in Zanzibar settings.

As a household strategy, aquaculture (constituting mostly seaweed farming) was found to be concerning in its monetary returns and poverty incidence. Households pursuing this strategy were universally earning under the poverty line, with incomes significantly lower than other strategies. Incomes although very low tended to be stable. These households also were more likely to be headed by women, and also older age groups—with this latter likely explaining the clustering of aquaculture and remittances/pensions together as a single strategy in the HCA. The results here reiterate past findings that seaweed farming provides a small but relatively stable income, but is likely insufficient to break poverty traps (Torell et al. 2017; Rimmer et al. 2021). Furthermore, the continuing stability of this income is uncertain in the face of ongoing climate stressors and disease breakouts (Hassan and Othman 2019; Largo et al. 2020). Regarding climate change, seaweed farmers were also found having low capacity for avoiding harm by adjusting to climatic changes, i.e. adaptive capacity, which is a serious aspect to be considered (de la Torre-Castro et al. 2022). This includes, for example, low organisational capacity, financial assets and mobility. Importantly though, the ‘low but stable’ narrative may not apply to seaweed farming as a household livelihood strategy, compared to when seaweed farming functions as a supplementary income within households. This represents a key finding in that the role of income from seaweed farming in the household is important. When seaweed farming is the dominant livelihood within households, these households have universal poverty incidence compared to households where seaweed farming may be providing supplementary income alongside the higher incomes generated from fisheries or land-based livelihoods, Income and poverty incidence within Zanzibar seaweed farming has mostly considered the individual-scale or as contributive incomes to households (e.g. Msuya 2011; Fröcklin et al. 2012), so we therefore emphasise the importance of viewing the livelihood in relation to the household-scale and its conjunction with other livelihoods within that household.

For those with continuing reliance on seaweed farming, initiatives that seek to increase quality and yields and thus increase income are a priority (Largo et al. 2020; Charisiadou et al. 2022; Makame 2022). To overcome challenges of low yields from exposure to temperature variation in shallow depths, innovative methods of farming seaweed in deeper water have been explored (Brugere et al. 2020). This strategy has shown to be empowering for those participating, although it is constrained by gender norms which prevent women from learning skillsets such as swimming and boat handling (Brugere et al. 2020). Investment in developing improved seed banks and varieties may be another route to developing resilience in the sector (Matoju et al. 2022). This could particularly be an avenue to explore regarding external funders. The large exits from seaweed farming also demonstrate that this currently is not the dominant main livelihood for coastal women across Zanzibar (small-scale business being the dominant). The current situation in Zanzibar shows the importance in monitoring how livelihoods are temporally transitioning in highly dynamic environments, as here a fairly dramatic shift has been demonstrated in just a 6-year period. Historically, seaweed farming has gone hand-hand in with women-focused initiatives by MPAs (e.g. establishing seaweed farming committees) due to its prevalence (Gustavsson et al. 2014). It may be helpful now to broaden actions to include the emerging main livelihoods, namely fisheries and small-scale businesses, and continue monitoring for future change. The need to consider historical trends and ongoing transitioning has been demonstrated elsewhere in tropical seaweed farming contexts, with emphasis that the introduction of seaweed farming must be followed up with insurance that it can be maintained over time to support people’s well-being and disenable poverty traps (Steenbergen et al. 2017; Andréfouët et al. 2021). This can also apply to other introduced livelihoods from interventions in order to enable long-term positive impacts and is a key recommendation for MPAs and wider coastal development contexts.

Across the 6-year period, the dominant main livelihood remained as fisheries for men, although exits occurred in MIMCA and the extended MBCA boundary. Comparatively, these exits were minimal in the MBCA. Land-based livelihoods, particularly agriculture, livestock and forestry and work within both the informal and public sectors were becoming increasingly common. Specific factors that may have influenced remaining or exiting fisheries are beyond the study’s scope. However, more widely in the region, these choices are known to be impacted by various socioeconomic factors including degree of specialisation within fishing, market accessibilities and institutional support (Daw et al. 2012). These factors can be highly place-specific, differ depending on scale and integrate with wide systemic factors such as local geographic contexts, and thus, generalisations should be avoided. MPAs may play a role, possibly through institutional support or regulations that may induce exits. Illegal fishing, e.g. use of beach seines, drag nets, etc., continues to be a persistent problem and grievance for compliant fishers in Zanzibar (Wallner-Hahn et al. 2016). Possibly, better enforcement of fishing regulations in the MBCA contributes to lower exits compared to the lesser enforced MIMCA, but this would need further exploration.

Despite apparent exits, the most common livelihood strategy at the household-scale fisheries was found to be fisheries. The strategy had higher monetary returns than other strategies through higher per capita income, lower proportion of households earning under the poverty line and stochastic dominance—except in relation to tourism strategies. However, multidimensional poverty was not significantly lower than the other strategies, except for aquaculture/other (which was significantly higher than all others). Therefore, although fishery strategies appeared higher returning monetarily, this has not necessarily gone hand in hand with lower deprivation across the education and basic infrastructure domains used within the MPM. Speaking more broadly, higher income does not always translate to, or substantially change, relative terms of poverty alleviation in fishery contexts, including wealth, capabilities or social inclusion (Béné and Friend 2011). Importantly, small-scale fisheries largely exist within rural economies and can be prone to the general constraints that affect wider rural sectors such as limited infrastructure and political underrepresentation (Béné 2006). Leading on from that, it is a possibility that similar constraints exist across the different strategies, including fisheries, land-based livelihoods and tourism. These constraints can lead to for example reduced capabilities in turning income into assets and may point to a need to consider fisheries within the wider rural development sphere alongside, e.g. agriculture, as opposed to coastal and marine management spheres alone.

Conversely to men, women were increasingly entering into fisheries, with this seemingly being a common destination from seaweed farming exits. As women increasingly engage with various fishery livelihoods, and to cope with growing gleaning fisheries following seaweed farming exits, now more than ever is gender awareness within fishery management urgently needed. Whether there is competition over certain intertidal species, such as octopus or sea cucumbers, which may be harvested by both men and women, would be interesting to further explore. Where the MPAs in context are concerned, examples of gender-aware approaches could include involvement of diverse actors within all governance and management phases including design stages, decision-making and monitoring (Kleiber et al. 2018) and identifying norms and constraints that induct gender differences into livelihoods and the subsequent outcomes (Lawless et al. 2019; Stacey et al. 2019). Engaging with and fostering women-led fishery initiatives and networks can also play key roles (Galappaththi et al. 2022). Ideally, adaptive management approaches to gender sensitivity that work within a fast changing social-ecological environment should be taken, as described previously in Zanzibar (Fröcklin et al. 2013). Building collaborations within the development sector, particularly with actors whom have worked successfully with gender approaches, may also be valuable (Mangubhai et al. 2022). This latter point may be particularly relevant when coordinating poverty alleviation and fishery management objectives within MPAs.

As tourism livelihood opportunities and economic benefits are a key part of the narrative surrounding community benefits from MPAs—these livelihoods were of particular interest in the study regarding their monetary returns in relation to more longstanding livelihoods such as fisheries. The high variance of incomes within tourism-orientated households indicates that this too is not a reliable strategy for maintaining a decent income. The differential access to this livelihood between men and women has also been highlighted as a potential contributor to gender division in the benefiting from MPAs (Pike et al. 2022). Households within the extended MBCA boundary were more likely to pursue tourism strategies than households within the other MPAs. Increases in tourism for both men and women’s main livelihoods were also observed in the extended MBCA boundary across the 6-year period. This is likely explained at least in part by this area being a longstanding popular location for tourists, regardless of the MBCA presence. Overall though tourism being a household livelihood strategy, as well as an individual livelihood, was relatively uncommon. This is particularly notable with its rarity in the MBCA, which due to its being the oldest, one may expect to see some indication of increasing tourism employment. Possibly, employment in hotels, restaurants and similar, is outsourced from local villages and/or constraints exist related to vocational training and language skills necessary for higher earning roles in the sector (Lange 2015). From a poverty alleviation standpoint, it therefore should not be assumed that this strategy offers a better alleviation potential than traditional fishery livelihoods. Importantly, fishery livelihoods must not be excluded in instances when tourism is a regional economic priority—with this having wider relevance to poverty alleviation, sustainable development and blue growth agendas.

In this study, the focus centred on monetary returns from livelihood strategies as a major contribution from livelihoods towards people’s well-being, and wider poverty alleviation contexts. This is not to say that monetary returns are the only motivation for pursuing different livelihoods. For example, fishing livelihoods are widely described to contribute to people’s identities, ways of life and engagement in social and cultural norms, all of which motivate non-monetary reasons for pursuing or remaining within these livelihoods (e.g. de la Torre-Castro and Lindström 2010; Cinner 2014; Santos 2015). In terms of tourism, cultural and social factors such as taboos concerning working with alcohol or wearing specific uniforms may inhibit some from working in the sector—including clashes with gender norms and expectations (Maliva et al. 2018). Furthermore, which activities are culturally deemed as suitable for people of different genders can lead to adherence to traditional activities (de la Torre-Castro et al. 2017), and a wider societal shift would be necessary to change this. Although beyond the scope of the study, the broader picture of why livelihoods are pursued and how they are valued is important to consider. This study also did not investigate intra-household differences, such as income allocation across members. These aspects are a key area of gender difference, which can importantly contribute to uneven poverty distribution within households (Vijaya et al. 2014).

## Conclusions

This study reiterates other recent findings (e.g. Eriksson et al. 2020; Roscher et al. 2022a) in showing that assumptions surrounding rural coastal livelihoods should be tested, as realities may differ. In this context of Zanzibar’s MPAs, it was found that livelihoods are highly dynamic and for women there has been an almost entire shift away, within 6 years, from seaweed farming into small-scale business and fisheries. This indicates that livelihoods should be monitored for change, in order to inform effective management, with this applying to wider contexts. Regarding seaweed farming, this was found to be most adopted by women-led households, particularly amongst older groups. The study also showed a need to re-consider the narrative surrounding income from seaweed farming as being low, but stable, and contributing to households. If seaweed farming is the main income stream in the household, this is a separate situation and the households where this applies, should be prioritised in poverty alleviation mechanisms. The study also challenges the notion that tourism offers higher monetary returns than more traditional livelihoods (such as fishing or land-based alternatives), as neither income nor poverty incidence was significantly different across MPAs. Following that, tourism did not demonstrate a benefit for MPA residents in this regard. As fisheries remained the most common livelihood, both individually for men, and more widely as a household strategy, means of alleviating poverty within this sector could be widely beneficial. It would be important to consider the multidimensional domains, alongside income, and we’d suggest further exploring how fisheries can be further integrated into the rural development sphere, in Zanzibar and similar contexts. Finally, as women increasingly engage in fishery livelihoods, it should be emphasised that gender-aware fishery management and governance that is adaptive to dynamic livelihood and gender norms is a key recommendation for MPAs.

### Supplementary Information

Below is the link to the electronic supplementary material.

Supplementary file1 (PDF 125 KB)
