# Supplementary material for: Dynamic livelihoods, gender and poverty in marine protected areas: Case study from Zanzibar, Tanzania
Source: Ambio. 2024 Apr 22;53(8):1218–33. doi: 10.1007/s13280-024-02010-x (PMC11183001; doi:10.1007/s13280-024-02010-x)
Supplement: Supplementary file 1 — Supplementary file1 (PDF 125 KB) [file 13280_2024_2010_MOESM1_ESM.pdf]

Ambio

Supplementary Information
This supplementary information has not been peer reviewed.

Title: **Dynamic livelihoods, gender and poverty in marine protected areas: case study from Zanzibar, Tanzania**

Table S1

Sub-categories of livelihoods used to define the eventual six livelihood main categories.

Fishery Sector	Aquaculture Sector	Agriculture, Forestry and Animal Husbandry	Tourism	Public Sector	Informal Sector
Fishing	Seaweed farming	Crop cultivation	Restaurant work	Police	Baking
Fish trading	Seaweed processing	Livestock rearing	Tour guide	Soldier	Tailoring
Gleaning	Other aquaculture	Firewood collection	Hotel work	Doctor	Grocery store clerk
Octopus fishing		Charcoal production	Driver	Teacher	Making clothes
Making nets			Boat operators	Madrasa teacher	Construction
Making derma traps			Dolphin tours	Government worker	Knitting
Fish vending				Civil servant	Small business
Selling octopus				Fisheries Officer	Selling chapati
Drying anchovies				Engineer	Selling juice
Fixing nets					Soapmaking
Fish auctioneer					Handicrafts
Crab fishing					Selling produce
					Neighbourhood watch
					Thatch making
					Dala-dala conductors and drivers
					Petrol station work
					Welding
					Babysitting
					Healers
					Sales representatives
					Stone crushing (kokoto)
					Beach vendours

Table S2 Descriptions of livelihood sectors. A list of the livelihood sub-categories which classified within the sectors is available in Table S1.

**Sectors**	**Description**
Fisheries	Livelihoods related to all aspects of the fishery sector including both fish and invertebrates. This includes roles in the pre- and post-harvest chain such as making nets and dema traps, drying catches and fish vending.
Aquaculture	Livelihoods related to all aspects of aquaculture, which in Zanzibar predominantly involves seaweed farming including work within post-harvest processing. Other forms of aquaculture in Zanzibar include half-pearl production and farming of various fish and invertebrates (Charisiadou et al. 2022).
Agriculture, Forestry and Livestock	Livelihoods related to all aspects of agriculture including crop cultivation, harvesting, and animal husbandry. In addition, livelihoods related to forest-derived natural resources including firewood collection and charcoal production.
Tourism	Livelihoods related to employment within tourism-related roles, including work in hotels, restaurants, tour operations, and dive centres. This grouping only includes those with employment, either formal or informal (e.g. non-contracted roles), and does not include individual enterprises or small businesses aiming at the tourist economy, for example beach vendors or market sellers.
Public Sector	Livelihoods related to formal, salaried employment within the public sector and any aspect of government work. Employment in these roles imply employee benefits such as pensions and annual leave.
Informal Sector	Livelihoods related to casual labour and small businesses. This includes trades such as construction, textiles work or welding, selling of goods, work in shops, or transportation (e.g. *dala-dala* conductors or petrol pump operators). These livelihoods do not have formal employment or employee benefits, with income possibly varying without long-term contractual agreements.

Table S3

The World Bank Multidimensional Poverty Measure (MPM) used to assess poverty incidence in households across the livelihood clusters. Indicator description are constructed based on (World Bank 2018).

† Corresponding to compulsory primary school age in Tanzania.

Poverty Domain	Indicator (0/1 outputs)	Weight
Monetary	Daily income per capita is less than 1.9 USD	1/3
Education	Atleast one child of school age (>7†) up to or under age 14 is not enrolled in formal education	1/6
	Noone over the age of 15 has attained primary education	1/6
Access to basic infrastructure	The household lacks access to running drinking water	1/9
	The household lacks access to private sanitation facilities	1/9
	The household lacks access to electricity	1/9

Table S4

Household characteristics used as explanatory variables in the multinomial logistic regression model.

Household characteristics	Indicators	Data type
MPA situation	Situation within MBCA, the extended MBCA boundary or MIMCA	Nominal
Household size	Total number of household members	Discrete
Gender of household head	Woman as head of household (Yes/No)	Binary
Age of household head	Age in years	Discrete
Education of household head	Access to secondary education equivalent or above (Yes/No)	Binary

Table S5

PCA loadings on factors 1-5 with eigenvalues >1. The loadings provided the input for the hierarchal clustering analysis to obtain the livelihood strategies.

Livelihood sector	Factor 1	Factor 2	Factor 3	Factor 4	Factor 5
Fisheries	-0.550173294	-0.291006078	-0.271819855	0.286741049	-0.300068241
Aquaculture	-0.047872399	0.52641206	-0.308744259	-0.027505556	0.154042163
Agriculture, forestry and livestock	-0.044414106	-0.06133412	-0.031541005	0.068912379	0.908893878
Tourism	-0.045327607	-0.065694154	-0.050126402	-0.939501096	-0.069044824
Informal Sector	0.829603185	-0.160233022	-0.146119996	0.144670894	-0.14861069
Public Sector	-0.050862227	-0.043329818	0.889649399	0.039957283	-0.009674515
Other	-0.012726506	0.776257764	0.120234569	0.084175637	-0.182197861

Table S6

Post hoc Wilcoxon rank sum test showing pairwise differences in mean per capita income across the livelihood clusters (Kruskal Wallis: p = 3.27e-07).

	Fisheries	Tourism	Aquaculture/other
**Tourism**	0.65	-	-
**Aquaculture/other**	>0.005	>0.005	-
**Mixed land-based**	0.01	0.26	>0.005

Table S7

Post hoc Wilcoxon rank sum test showing pairwise differences in World Bank multidimensional poverty measure (MPM) scores across the livelihood clusters (Kruskal Wallis: p = 0.0001).

	Fisheries	Tourism	Aquaculture/other
**Tourism**	0.83	-	-
**Aquaculture/other**	0.0006	0.003	-
**Mixed land-based**	0.40	0.56	0.002

References

Charisiadou, S., C. Halling, N. Jiddawi, K. von Schreeb, M. Gullström, T. Larsson, and L. M. Nordlund. 2022. Coastal aquaculture in Zanzibar, Tanzania. *Aquaculture* 546: 737331. doi:10.1016/j.aquaculture.2021.737331.

World Bank. 2018. *Poverty and Shared Prosperity 2018: Piecing Together the Poverty Puzzle.* Washington, DC: World Bank. License: Creative Commons Attribution CC BY 3.0 IGO.
